# Use of Self-Generating Foam Gel Composition with Subsequent Injection of Hydrogel to Limit Gas Inflow in Horizontal Wells of Vostochno-Messoyakhskoye Field

**DOI:** 10.3390/gels10040215

**Published:** 2024-03-22

**Authors:** Aleksey Telin, Dmitriy Karazeev, Sergey Vezhnin, Vladimir Strizhnev, Aleksey Levadsky, Anton Mamykin, Lyubov Lenchenkova, Ravil Yakubov, Alsu Fakhreeva, Alfir Akhmetov, Aleksey Oleynik, Anton Shirobokov, Bulat Minnebaev, Ilyas Mullagalin, Ramil Bakhtizin

**Affiliations:** 1 Ufa Scientific and Technical Center, LLC., 99/3, Kirov Street, 450078 Ufa, Russia; 2 Faculty of Mining and Petroleum, Ufa State Petroleum Technological University, 1, Kosmonavtov Street, 450064 Ufa, Russia; 3 Ufa Institute of Chemistry, Ufa Federal Research Center, Russian Academy of Sciences, 71, Oktyabrya Avenue, 450054 Ufa, Russia; 4 Mavlyutov Institute of Mechanics, Ufa Federal Research Center, Russian Academy of Sciences, 71, Oktyabrya Avenue, 450054 Ufa, Russia; 5 Messoyakhaneftegaz JSC, 77, Kholodilnaya Street, 625026 Tyumen, Russia; 6 Academy of Sciences of the Republic of Bashkortostan, 15, Kirov Street, 450008 Ufa, Russia

**Keywords:** horizontal wells, gas inflow control, self-generating foam gel composition, hydrogel

## Abstract

Gas inflow control in oil wells is one of the most challenging types of repair and sealing operations, the success rate of which does not exceed, as a rule, 30%. Conventional shutoff methods are often ineffective for this purpose. For instance, cement solutions cannot be injected into wells in the required volumes, while gel screens can only temporarily block the breakthrough zones, as gas easily seeps through the gel, forming new channels for gas inflow. Technology for the two-stage injection of gas-insulating gel systems for gas control in horizontal wells was developed. At the first stage, a self-generating foam gel composition (FGC), consisting of gel-forming and gas-forming compositions, was used. A foam gel structure with enhanced rheological and flow characteristics was formed over a controlled time as a result of the interaction between the gel-forming and gas-forming compounds. A PAM-based hydrogel crosslinked with an organic crosslinker was added to the FGC at the second stage of treatment. The laboratory experiments substantiated the technology of well gas and water shutoff by the sequential injection of self-generating foam gel composition and hydrogel. Field tests confirmed the correctness of the chosen concept. It is very important to clearly identify the sources of gas inflow for the success of this well intervention and take into account the well design, as well as the reservoir geological structure and characteristics. The gas shutoff operation can be properly designed for each well only by comparing all these factors. The validity of the selected technology was tested through a series of laboratory experiments. Successful laboratory tests allowed for the application of the studied technology in a field setting, where the gas shutoff agent was injected into three horizontal wells. As a result of the field application, the gas inflow was successfully isolated in two wells. However, the application of the technology failed in the third well which gave an opportunity to revisit the technology’s design and to review the sources of gas inflow. Overall, the achieved success rate of 66% demonstrated the high efficiency of the studied technology and supported its wider application in the field.

## 1. Introduction

The development of complex formations with a gas cap determines the increasing need for repair and sealing operations for gas inflow control. Intensive gas breakthroughs complicate oil recovery. The practice of repair and sealing operations with cement solutions shows that during their injection, there is a sharp increase in pressure up to the values comparable to those of the column pressure test. This increase in pressure does not allow the required volume of cement to be injected for the effective sealing of water and gas inflow channels into the well [1]. It should be noted that gas inflow control operations in horizontal oil wells are among the most challenging types of repair and sealing operations with the lowest success rates. The use of conventional hydrogels for these purposes, which are applied for water [2] and gas [3] shutoff, is usually not sufficiently effective since the gas easily penetrates the gel structure and forms new gas inflow channels. A new promising direction for gas shutoff is the use of nanosilica. The gas isolation screen is formed from nanosilica gel, which transforms into a glass-like body in the formation. This approach allows for controlling gas inflow in wells drilled in dense rock formations [4,5,6]. Considering that the object of our study, the PK_1−3_ formation of the Vostochno-Messoyakhskoye field, is a weakly cemented sandstone, this approach does not apply to it. Foam [7,8,9], polymer foam [10] and foamed gel [11] compositions provide more effective control of gas breakthroughs. The breaking-through gas, when interacting with a surfactant solution, forms a foam, which in the polymer solution turns into a foam polymer system with increased structural, mechanical and, accordingly, gas-sealing properties. One way to improve the gas isolation efficiency of the surfactant solution’s foaming rim is by reinforcing it with a gel-forming rim [12]. 

A composition of a biopolymer with non-ionic surfactants has also been used [13] to prevent gas kicks during well killing, and it is possible to use a foam gel consisting of a crosslinked polymer, as well as gas-releasing and foaming agents for the same purposes [14].

It should be noted that in recent years, foamed gel compounds have found a variety of applications in oil production processes. Thus, foam gels have been successfully used for conformance control in both low-permeability [15] and high-temperature [16] reservoirs. Wormholes in loose sandstones are sealed using foams [17]. The advantage of this approach, according to article [17], is the reduction in the consumption of the sealing composition and, accordingly, the reduction in the workover costs, which is especially important for horizontal wells. Papers [18,19] describe foams for water shutoff in fractures in carbonate reservoirs. A heat-resistant foam gel based on phenolic resin and alkaline lignosulfonate has even been used to control the vapor-cyclic effect [20]. The use of self-generating foam gel composition (FGC) provides the strongest process benefits since their injection does not require a compressor and coiled tubing equipment. Nitrogen gas is generated due to the redox reaction of inorganic salts—sodium nitrite and ammonium chloride; non-ionic surfactants are used as foaming agents, and hydrogel is produced from partially hydrolyzed polyacrylamide crosslinked with an organic acid aluminum salt [21]. Such foams are used both for conformance control and for water shutoff. The article [22] emphasizes that FGC has a higher gas migration control ability than the foam polymer system. The authors of the work [23] proposed a double-crosslinking foam for water shutoff and well killing. Two-mesh foam gel is also proposed to control gas breakthroughs in the case of water-alternating-gas injection in fractured cavernous reservoirs [24].

Foamed gel compositions, which are based on partially hydrolyzed polyacrylamide (PAM) crosslinked with chromium ions (Cr^3+^) in combination with surfactants that were foamed with nitrogen, are widely used for water shutoff purposes. A self-generating foam gel composition was used for water shutoff in the Soviet Union. It also consisted of partially hydrolyzed PAM crosslinked with Cr^3+^ ions, with the addition of anion-active surfactants. Nitrogen is generated in this composition due to the reaction of sodium nitrite with ammonium chloride during the acidification of the solution with hydrochloric acid [25]. Essentially similar technical solutions using self-generating foam gel systems were patented [26,27,28]. A gas-filled composition for water shutoff is known, which is a stable foam gel emulsion stabilized by the emulsifier on the interface of the phases both from the hydrocarbon (dispersion) medium side and by a crosslinked polymer in the form of its emulsion in the oil from the dispersed phase. The interaction of the components of the composition produces a surface-active gas-filled gel that firmly holds the gas generated by the reaction of sodium nitrite and ammonium chloride. In addition, the composition contains a dispersed filler of submicron size [29].

The original approach to water and gas shutoff using foam polymer systems in fractured formations was shown by the authors of article [30]. The purpose of this study was to increase the efficiency of foam compositions by using polymer chemicals and various types of surfactants. A gas-generating system of inorganic salts was used as a gas source. Experiments showed that the use of a foam gel composition is more effective in large fractures (0.15–1 mm) than the use of gel systems alone. At the same time, the residual resistance factors (Frr) were 10–100 units, and the composition carryover gradients ranged from 3 to 50 atm/m. The commercial application of foam gel systems, which are based on surfactants and hydrogels, for water and gas shutoff in fractured reservoirs in different regions of the world has confirmed foam gel systems’ high efficiency [31].

The purpose of this study is to experimentally substantiate the gas inflow control technology and its field confirmation using foamed gel and hydrogel compositions.

## 2. Results and Discussion

### 2.1. Laboratory Tests 

The study of the properties of FGC unexpectedly revealed that an increase in temperature to 60 °C practically does not affect the foam expansion ratio and the half-life of FGC could not be determined at all. The fact is that the structural and mechanical properties of gels in lamella films are so high that the resulting foam practically does not collapse (like solid foams).

**Figure 1:**
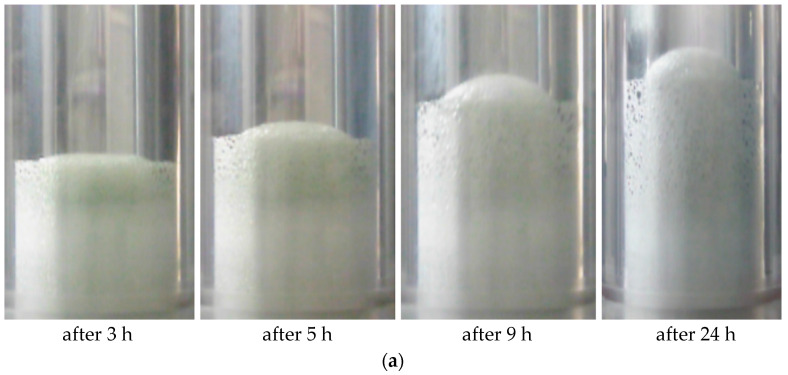

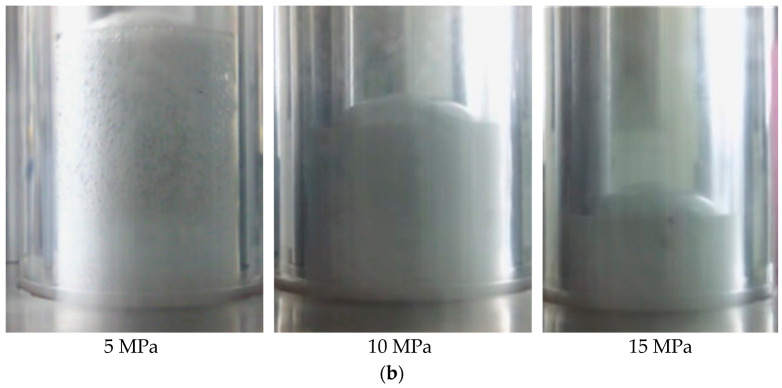
The formation of FGC in the Micro PVT apparatus (**a**) at different durations and pressures of 5 MPa; (**b**) at different pressures after 24 h.

Experiments conducted using the Micro PVT apparatus showed that the increase in foam volume stops after 24 h at a pressure of 5 MPa (Figure 1a).

The pressure has an expected impact on the foam expansion ratio of self-generating FGC, namely, the foaming capacity of two-phase foam systems significantly decreases even with a slight increase in pressure. The foam structure failed to form in the fresh water when the pressure was increased to 2.5 MPa, given the following composition of FGC: [PAM A523 (0.6%) + sodium nitrite (2.7%) + ammonium chloride (2.3%) + chromium acetate (0.3%)]. It was possible to increase the foam expansion ratio to a value above 8 only in case of an increase in the concentration of gas-generating salts in the composition by 3 times (pressure 2.5 MPa). The foam expansion ratio decreased as follows with a further increase in pressure: to 3 at 5 MPa; to 2 at 10 MPa and to 1.5 at 15 MPa (Figure 1b).

It should be noted that the foaming time and the time of loss of fluidity for compositions with a high content of the gas-generating mixture should provide sufficient time for injecting compositions into the isolated interval before crosslinking. The acceptable time for this process ranges from 2 to 6 h, and the composition should maintain a sufficient foam expansion ratio. In this study, the fluidity loss time ranged from 4 to 6.5 h.

**Figure 2:**
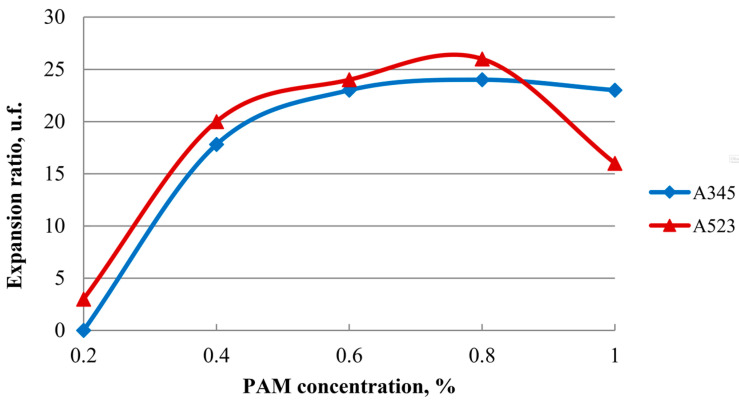
Dependence of the foam expansion ratio on the polymer concentration.

As can be seen from Figure 2, compositions with a polymer concentration of 0.6% for both high-molecular-weight PAM (A345) and low-molecular-weight PAM (A523) have the necessary foam expansion ratio parameters. This is because both high- and low-molecular-weight polyacrylamides form a crosslinked polymer structure called a hydrogel. At this polymer concentration, the necessary structural and mechanical properties of the hydrogel are provided, which are manifested in the same foam expansion ratio parameters.

**Figure 3:**
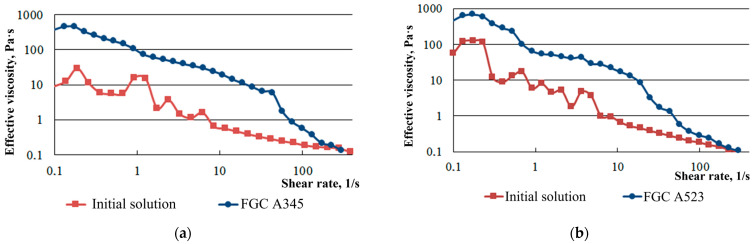
Dependence of the effective viscosity on the shear rate for FGC prepared on the basis of (**a**) PAM A345; (**b**) PAM A523.

The results of rheological studies performed using the method in [32] showed that the viscosity of FGC significantly exceeds the viscosity of the non-foamed gel (Figure 3).

It should be noted that immediately after the mixing of foam components, the foam system has low effective viscosity values, which allows it to be delivered into the reservoir. The viscosity of the composition significantly increases at the time of its final crosslinking, which meets the requirements of effectively sealing the gas inflow filtration channels.

Oscillating stress tests (dynamic tests) allow the study of the viscous and elastic reactions of a liquid sample depending on the rate of impact on it, in other words, constructing the dependence of oscillating stress or strain on a given angular velocity or frequency.

**Figure 4:**
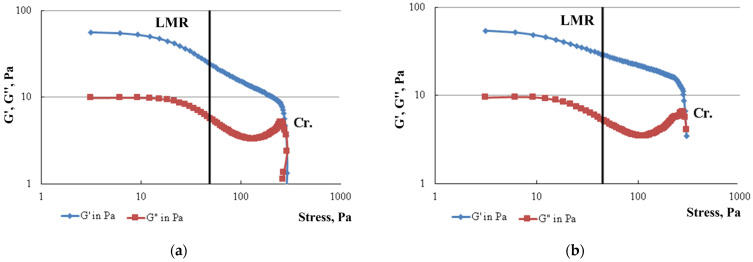
Dynamic oscillation tests of FGC based on (**a**) PAM A345; (**b**) PAM A523.

Figure 4 shows the results of the dynamic measurements of FGC samples, which provide information about rheological properties, due to the possibility of separating the elastic and viscous components.

The elastic modulus (accumulation modulus) G′, Pa characterizes the accumulated strain energy in the system and reflects the characteristics of the sample as a solid (elastic component). The value of the viscous modulus (loss modulus) G″, Pa determines the energy dissipation and the behavior of the sample as a liquid (viscous component). The range of linear viscoelastic behavior (linear measurement range—LMR) is the area where both curves G′ and G″ have an area of constant values in the form of a plateau. This is the range of strain values in which the structure of the test sample does not collapse due to strain. The point of intersection of the modulus of elasticity and viscosity or the point of failure of the structure (crossover—Cr.) is the point of equality of the modulus of elasticity G′ and viscosity G″. Viscous behavior prevails over viscoelastic behavior after this point.

The studied samples of FGC—PAM A345 and PAM A523—are characterized by a small range of linear viscoelastic behavior. In both cases, no significant differences were found in determining the linear measurement range, the value of which would be more than 50 Pa. The ratio of the viscous component to the elastic component in the presence of both PAM A345 and A523 is approximately 4.5. However, the highest stress or strain value corresponding to the intersection point of the elastic and viscosity moduli (the crossover point), after which the transition of FGC to a viscous state occurs, is characteristic of a composition containing a sample of low-molecular-weight PAM. Therefore, the crossover point is 295.0 Pa in a composition containing PAM A523 and 285.7 Pa in the case of PAM A345 (Table 1).

It should be noted that both the modulus of elasticity and the modulus of viscosity decrease when stress values exceed 30 Pa. This is likely attributed to the initiation of structural failure in foam systems, including the collapse of part of the cells in the formed foam gel. The complex modulus G*, Pa can be calculated using the value of the applied stress and the resulting strain: $\left|G*\right|=\sqrt{{G^{\prime }}^{2}+{G^{\prime \prime }}^{2}}$.

**Figure 5:**
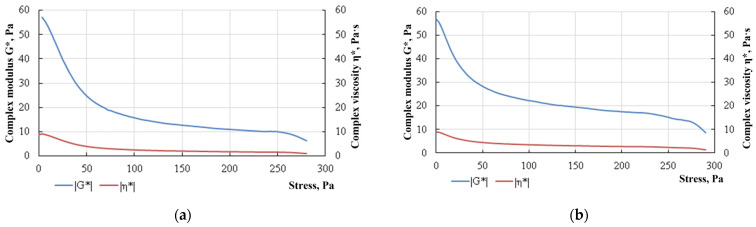
Stress-induced change of the complex modulus and viscosity of FGC based on (**a**) PAM A345; (**b**) PAM A523.

The corresponding measurements show that the values of the complex modulus G* in a composition with a low-molecular-weight PAM A523 are 1.6–1.8 times higher in the case of the stresses ranging from 50 to 250 Pa. This indicates greater stability of foam lamellae, which is manifested by the resistance of the FGC to external deforming forces through the recovery of the original shape (Figure 5).

The viscosity measured in the oscillation experiment is called complex by analogy with the complex modulus. The complex viscosity includes an elastic component and an element similar to viscosity under steady flow conditions: η* = G*/ω.

**Figure 6:**
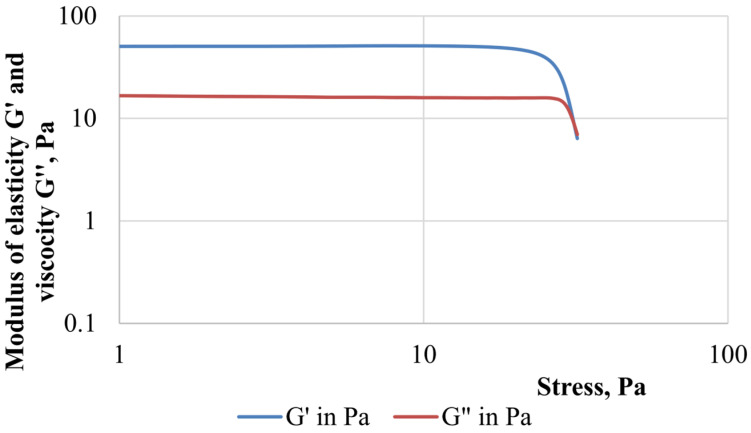
Oscillatory rheometry of a hydrogel.

Gas shutoff operations often employ a technological technique when the bulk of the grouting material (for example, hydrogel) is supported by a more rigid composition such as cement or curing resin [1]. In this study, a fairly rigid hydrogel containing 1.7% PAM grade A345, 0.6% paraform and 0.2% resorcinol was chosen. This hydrogel composition has been successfully used for water-shutoff activities [33]. A comparison of the elastic modulus of FGC and hydrogel showed that the elastic modulus of a hydrogel crosslinked with a complex organic crosslinker exceeds that of FGC: 4199 Pa for hydrogel and 3585 Pa for FGC. The dynamic definitions of the modulus of elasticity and the modulus of viscosity are provided in Figure 6, which shows that the region of viscoelastic behavior is constant over a large range of applied stresses.

Consider that the gas shutoff properties of FGC were determined in previous studies [34], in which it was shown that the foam gel efficiency is many times higher than that of the foam polymer and hydrogel. Therefore, in this study, the water shutoff properties of FGC were clarified, and filtration experiments were conducted in the porous media of different natures and structures. The residual resistance factor (Frr) was chosen as the efficiency parameter; it was found that Frr has the highest value in the fracture and the lowest value in the sandstone (Table 2). Perhaps this is due to the selectivity of the effect of gels on large pores and fractures [2].

Consequently, self-generating FGC has water-isolating properties selective in permeability, the degree of manifestation of which depends on the size of the channels of the porous medium, which should be considered when selecting candidate wells for repair and sealing operations, as well as when determining the volume of the injected composition and the time of subsequent development of the well.

A hydrogel based on PAM, resorcinol and paraform was selected to reinforce the FGC, which has the necessary structural and mechanical properties [35] and successfully passed field testing under various geological and physical conditions [33].

### 2.2. Field Test

The work was carried out in three horizontal production wells with production casing diameters of 146, 168 and 178 mm, respectively. The wells are equipped with strainers and perforated filter liners, with a horizontal section length from 600 to 1500 m. Liner sizes for the wells under consideration are presented in Table 3.

**Figure 7:**
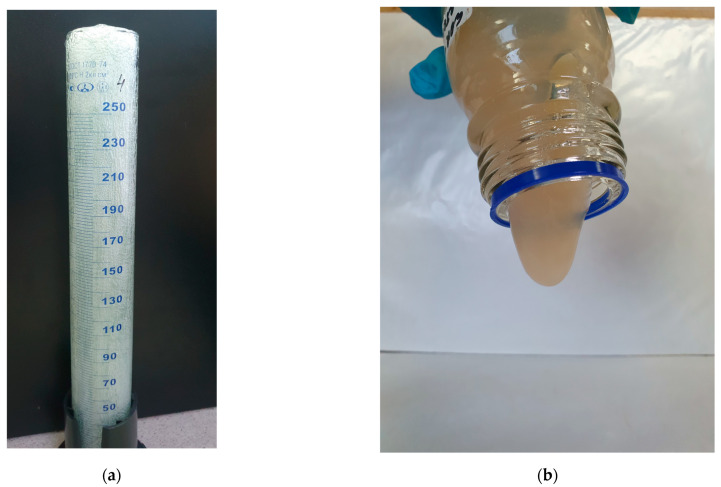
Photos of sample compositions: (**a**) self-generating foam gel composition; (**b**) hydrogel.

A distinctive feature of the use of FGC in field conditions is the addition of gas-generating components during the mixing of the composition at the wellhead with the generation of foam directly in the wellbore and in the bottomhole zone of the formation. Free nitrogen gas is released as a result of the interaction of the components of the gas-generating mixture, and the nitrogen bubbles are distributed in a viscous polymer solution. Moreover, the times of gas generation and polymer crosslinking are regulated depending on the reservoir temperature by varying the concentration of the components and acidifiers. Before injection, a qualitative determination of the properties of the FGC and hydrogel was performed. Figure 7 shows photographs taken just before injection. It can be seen that FGC (Figure 7a) is formed in the entire volume of the measuring cylinder, and the mature hydrogel shows a tongue from the bottle (Figure 7b).

A total of 100 m^3^ of FGC and 20 m^3^ of hydrogel with an organic crosslinker was injected in each well.

#### 2.2.1. Well W1

Free gas appeared immediately at the beginning of the well operation. According to the data of inflow profiling on 11 July 2020, the primary gas inflow was found in the interval of 1280—1370 m (68% of the gas inflow), the second inflow in the interval of 1680–1760 m (22%) and the third inflow (about 10%) below the current total depth of 1925 m. The first two intervals are the closest to the gas–oil contact: only 5 m along the vertical.

**Figure 8:**
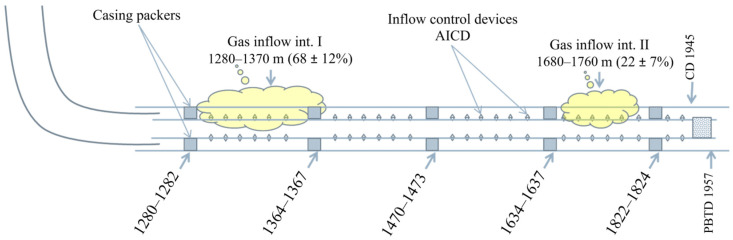
Design of well W1 with gas inflow sources.

Figure 8 shows the design of the well with the sources of gas inflow. Thus, the liquid flows from the reservoir into the well through the inflow control valves, which are arranged in such a way that they are opened in underbalance conditions. The control valves are closed in overbalanced conditions to prevent the contamination of the bottomhole zone of the formation during the flushing of the well. Therefore, it was necessary to perforate special openings in the liner during the repair and sealing operation at the top of the gas inflow intervals by pumping sealing compounds.

It was decided to perform the treatment of the well in two stages with the determination of the required volume of sealing compounds to limit gas inflow (Table 4). Each stage of treatment included, first, the injection of FGC to limit the gas inflow and then the injection of a viscoelastic hydrogel to reinforce the FGC and strengthen the created sealing screen.

Well W1 was shut-in for 24 h after each treatment stage. The well was shut-in after the second stage, and then the bottomhole was cleaned to the bridge plug at a depth of 1826 m. The bridge plug was not drilled, which reduced the length of the strainer by 119 m (by 18%). No polymer recovery was observed during the well flushing. This indicates its consolidation in the reservoir. The parameters of well operation (liquid rate Qliq, oil rate Qoil, watercut WC, bottomhole pressure Pbottom, dynamic level Hdyn, gas rate from gas cap Qg.c. and gas–oil ratio GOR) before and after gas shutoff treatment are presented in Table 5.

Table 5 shows that the inflow of gas from the gas cap was practically stopped as a result of the treatment. That effect lasted for more than a year. Thus, the gas breakthrough from the gas cap was stopped in well W1. In addition, it was found that the created gel screen can withstand extremely low bottomhole pressures (27 atm) for an extended period.

It is known that the inevitable clogging of the productive reservoir occurs during repair and sealing operations, along with the remediation of water and gas entries, which often results in a reduction in the oil flow rate. Therefore, it was proposed to perform hydrochloric acid treatment to recover the liquid flow rate to the pre-treatment-level values, followed by the drilling of the bridge plug at 1826 m to increase the drainage area. However, this treatment was not implemented because of the fear of a new gas breakthrough.

#### 2.2.2. Well W2

**Figure 9:**
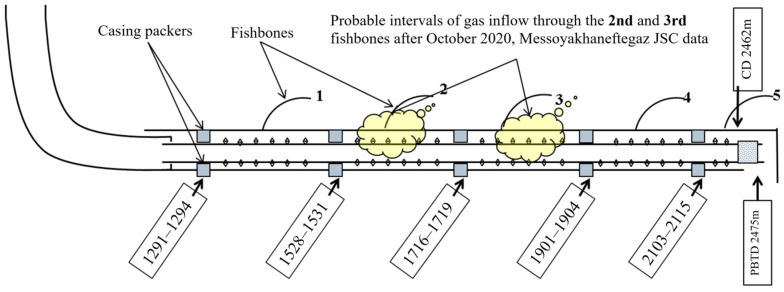
Diagram of design of well W2 with gas inflow intervals.

Free gas was found in well W2 fluid immediately after drilling. The well was shut-in on 9 August 2021 for inflow stimulation with hydrochloric acid. At the same time, the rate of gas inflow from the gas cap (Qg.c.) was approximately 20,000 m^3^/day. A rapid gas breakthrough occurred at the rate of 151,963 m^3^/day on 17 August 2021, during the well stabilization after inflow stimulation. A total of 20 m^3^ of hydrochloric acid (HCL) was injected during inflow stimulation between the swellable packers in the interval of 1700–2000 m (Figure 9) and, probably, the acid also entered into fishbones 2, 3 and 4.

Gas shutoff treatment was performed on 2 September 2021 in the interval of 1530 m (retainer packer)—1906 m (bridge plug) for the isolation of the gas inflow by the injection of 20 m^3^ of a PAM-based viscoelastic composition with a chrome cross-linker, followed by cementing with a volume of 7 m^3^. As a result, the cementing of the hole in the section of the second and third fishbones failed (the gas inflow from the gas cap was not completely isolated, and the isolation effect lasted only about one month). Well W2 had the following operating parameters at the date of the shutdown for well workover: Qliq—12.7 m^3^/day, Qoil—9.7 t/day, watercut—19.7%, Qg.c.—43,947 m^3^/day, dynamic level (Hdyn)—461 m, bottomhole pressure (Pbottom)—53 atm.

It was decided to inject sealing compounds using a foam gel composition reinforced with hydrogel in two stages (Table 6).

The technology of the mixing and injection of chemicals was similar to the operations carried out in well W1. The well was ramped up quickly and without complications.

Well W2 was commissioned on 16 September 2022 with the following parameters: Qliq—10 m^3^/day, Qoil—8.5 t/day, watercut—10.3%, Qg.c.—0 m^3^/day, Hdyn—376 m, Pbottom—46.93 atm. Table 7 shows that the gas inflow from the gas cap was completely stopped as a result of the field trial for 6 months until 25 March 2023, when the Qg.c. amounted to about 814 m^3^/day.

The operating parameters of well W2 as of 1 September 2023: Qliq—13 m^3^/day, Qoil—10.3 t/day, watercut—12%, Qg.c.—6329 m^3^/day, Hdyn—633 m, Pbottom—42.7 atm.

Thus, the gas breakthrough from the gas cap was isolated as a result of the activities in well W2. At the same time, the flow rates of oil and liquid did not change and the watercut somehow decreased, which indicates the selectivity of the effect of sealing compounds. As a result, it was found that the created screen can withstand extremely low bottomhole pressures (42.4 atm) for a long time.

#### 2.2.3. Well W3

**Figure 10:**
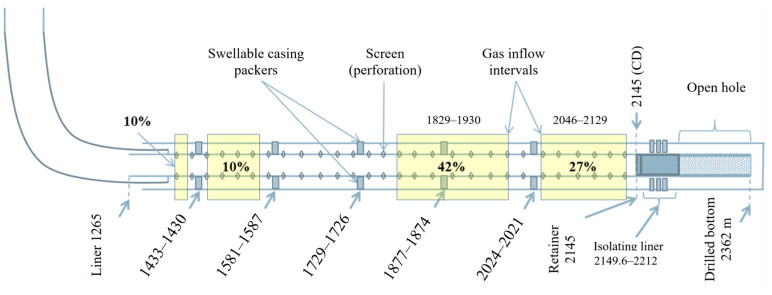
Diagram of the design of well W3 with gas inflow intervals.

The presence of an isolating blind liner in the interval 2149.6–2212.0 m is a specific feature of well W3 that distinguishes it from wells considered previously. Well W3’s design is shown in Figure 10.

An intensive gas breakthrough was observed starting from 14 June 2021, after well W3 was released from drilling: within a day, gas flow increased from 40,000 to 100,000 m^3^/day. The service contractor in October 2021 carried out repair and sealing operations to limit the gas inflow by shutting off the isolating liner in the interval of 2145–2221 m. The well was treated with a 30 m³ compound consisting of a PAM-based gel (PAM concentration—1.67%) and a chrome crosslinker after the packer retainer had been set at the depth of 2145 m. The gel treatment was followed by the injection of 4 m³ of cement. The reduction in gas flow rate (Qg.c.) lasted for a week, until 1 November 2021. After 5 November 2021, Qg.c. increased from 3000 to 26,000 m³/day, followed by a constant growth in Qg.c.

In order to plan the gas shutoff operations in the well, the inflow profile in the horizontal section of the wellbore was logged on 17–21 July 2023. An intensive inflow of gas from the PK1-3 formation in the interval of 1839–1935 m was observed in the dynamic mode according to the temperature logging data (according to the results of thermohydrodynamic modeling), and the share of gas inflow was 42% of the total inflow. A less intensive gas inflow, accounting for 27% of the total, was observed in the interval of 2046–2129 m. The gas inflow in the heel section of the well accounted for 20% of the total and corresponded to the intervals of 1310–1420 m and 1479–1573 m (Figure 10). The producing intervals of the reservoir were determined based on spectral noise measurement data.

To achieve reliable isolation of the gas inflow into the well, it was necessary to carry out repair and sealing operations to limit the gas inflow in the inter-packer (behind casing) intervals, which corresponded to 1729–1874, 1877–2021, 2024–2145, 1302–1430 and 1433–1578 m. However, there was a risk of reducing the oil flow rate due to the productive reservoir potentially clogging. According to the well logging results, the main gas inflow occurred at the interval of 1840–1850 m. As for other intervals, gas produced through them just accumulated in the wellbore. Consequently, the engineers at Messoyakhaneftegaz JSC decided not to isolate the intervals of 2024–2145 m (toe part) and 1302–1430 m, 1433–1578 m (heel part). In order to minimize the risk of oil production loss, the repair and sealing operations were carried out specifically to limit the maximum gas inflow (42%), corresponding to the inter-packer interval of 1729–1874 m. However, after isolating that interval only, there remained a risk of gas breakthrough from the gas cap through other untreated intervals identified by logging. The parameters of the well before the workover were as follows: Qliq—8.7 m^3^/day, Qoil—8.0 t/day, watercut—2.6%, Qg.c.—56393 m^3^/day, GOR—7045 m^3^/t, well liquid level—628 m, bottomhole pressure Pbottom—36.9 atm. The chemical treatment, similar to the treatments previously conducted on wells W1 and W2, was carried out in a single stage. The design of this operation is detailed in Table 8.

Well W3 was commissioned on 22 August 2023 with the following parameters: Qliq—15 m^3^/day, Qoil—12 t/day, watercut—15.3%, Qg.c.—3901 m^3^/day, Hdyn—676 m, Pbottom—37.5 atm.

Table 9 shows that as a result of gas shutoff, the flow rates of liquid and oil increased from 8.7 and 8.0 t/day to 12.6 and 10.1 t/day, respectively, while the watercut slightly increased: from 2.6 to 15%. The inflow of gas from the gas cap significantly decreased, but it began to rapidly grow after 2 September 2023 and amounted to 53,716 m^3^/day on 21 September 2023, i.e., it actually reached the gas flow rate value before the workover.

It seems that either the gas quickly bypassed the created gel screen and reached the neighboring intervals where gas inflow was also partially observed (according to the logging data) or it possibly broke through a leaky casing packer. These assumptions are also supported by that fact the breakthrough interval was close to the casing packer, i.e., the bypass distance was small.

### 2.3. A Comparison of the Results of This Study with Previously Published Data

Experimental and field studies confirm the results of several research groups on the advantages of FGC over polymer foams, foams and hydrogels for gas isolation [11,22,34]. It should be noted that the use of self-generating FGC with the subsequent injection of hydrogel for gas isolation is a new type of technology for gas flow restriction in oil wells. The duration of the effect on two successful wells is because the FGC does not contain surfactants and is not destroyed in contact with oil and water and the structural and mechanical properties of cross-linked polymers in the foam lamellae maintain its cellular structure for any length of time (the half-life of the foam could not be established). The high elastic modulus values of FGC along with the hydrogel allow the gas isolation screen to withstand extremely low bottomhole pressures when inducing flow into the well. One successful well showed a decrease in water cut, which confirms the conclusion obtained from the filtration experiments regarding the water-sealing properties of FGC.

## 3. Conclusions

The operations conducted to limit gas inflow in horizontal wells using technology based on self-generating FGC and hydrogel with organic crosslinking confirmed the correctness of the chosen approach.

The injected polymer was not detected in the produced fluid from wells W1, W2 and W3 after the gas shutoff operations, indicating its reliable consolidation within the formation. There was no gas breakthrough during the ramp up and subsequent operation of wells W1 and W2 after gas shutoff treatment, even at extremely low bottomhole pressures, which indicates the stability of the created screen over time. The period for achieving complete isolation of the gas breakthrough from the gas cap was approximately 16 months in well W1 and over 6 months in well W2. The increase in the gas flow rate in well W3 can be explained by the inflow of gas from the gas cap from other intervals or through a possibly leaky casing packer.

The novelty of this study lies in the application of self-generating foam gel compositions in combination with hydrogel for gas shutoff in horizontal wells. It should be noted that the foam gel system is formed without the addition of surfactants, and both gas formation from the system of inorganic salts and gel formation are synchronized in time.

The gas shutoff technology developed in the laboratory and tested on wells can also be successfully applied to water shutoff. To enhance the efficiency of gas shutoff in the future, the effect of pre-injecting a surfactant solution rim should be investigated. This operation may increase the duration of the effect because the released gas will form a foam system with the surfactant rim. Thus, the robust foam and gel barrier will be affected not by gas but by the created foam system, which will reduce the risks of dagger gas breakthrough. To strengthen the water-sealing barrier, the introduction of nano- and microdispersions into the final rim of the hydrogel could be explored.

## 4. Materials and Methods

### 4.1. Foam Gel Composition Production Method

The composition mixing method in the laboratory conditions included dissolving the polymer in fresh water for 30 min, as well as dissolving a gas-generating mixture consisting of ammonium chloride and sodium nitrite, with the addition of a crosslinker such as chromium acetate. The level of solution in a test tube during the study was measured just after the mixing of foam components and after 24 h. The foam expansion, solution stability and foaming time were determined. Polymers of grades A345 and A523 were used as partially hydrolyzed polyacrylamides. The molecular weights of these polymers and the hydrolysis degrees were 15 × 10^6^ Da and 15%; 3 × 10^6^ Da and 20%, respectively.

The hydrogel for the reinforcement of FGC was produced using polymer of grade A523 (1.7%), resorcinol (0.6%) and paraform (0.2%).

### 4.2. Visualization of Foam Gel Composition in Reservoir Conditions

The behavior of FGC at high pressures was studied using the Micro PVT device, which is an element of the SMP-SV system (Reservoir Modeling System, Visual Separator) (Kortekh, Mytishchi, Russia), which is provided with a polished sapphire tube at a pressure of 62.5 MPa.

### 4.3. Rheological Studies

The FGC viscosity curves were plotted using the rotary viscometer Haake Viscotester iQ (Thermo Fisher Scientific, Waltham, MA, USA). The measurements were carried out in shear rate control mode using a coaxial cylinder-type measuring cell with a cylinder diameter of 25 mm (CC25 DIN/Ti), which consists of two rotating and stationary coaxial vertical cylinders. A test composition is poured between the cylinders, which resists the rotation of the inner cylinder and transmits it to the outer cylinder. The shear stress and effective viscosity of the studied samples were measured with a variation in the shear rate from 0.1 to 300 s^−1^.

The modulus of elasticity and modulus of viscosity (G′ and G″) of FGC were measured using the Haake Mars III rheometer in controlled stress measurement mode using a plane-to-plane measuring system with a rotor and plane diameters of 60 mm (P 60/Ti). HAAKE Rheowin 4 Job and Data Manager rheological software (version 4.88.0002, Thermo Fisher Scientific, Waltham, MA, USA) allowed for conducting measurements and processing the results.

**Figure 11:**
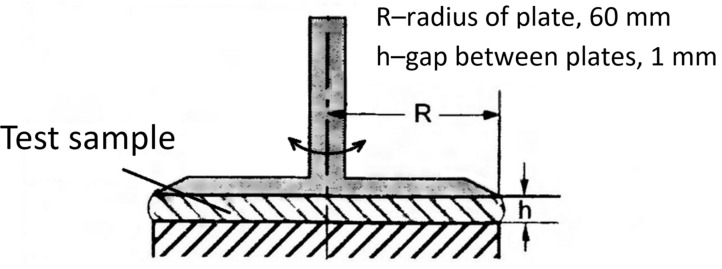
Plane-to-plane system; oscillation measurements.

A schematic of the perceptual elements in the oscillatory tests is shown in Figure 11.

Oscillation measurements were performed by a plane-to-plane system with a gap *h*. The disk is made of titanium to reduce the moment of inertia, which makes it possible to perform measurements up to frequencies of 100 Hz. In oscillation measurements at a frequency of 1 Hz, the amplitude of rotational oscillatory motion at the outer radius *R* of the disk is a fraction of a millimeter.

In our case, measurements were performed at an oscillation frequency of 1 Hz with tangential stress scanning τ (OSS—Oscillation Stress Sweep) from 0.1 Pa to the oscillation stall at τ of the order of 200 Pa. The tangential stress τ is set to rotate the disk, which is provided by the torque on the disk axis and is measured by the torque-setting current. The rotation angle is measured optically with very high accuracy. From the resulting angle of rotation at a given τ, the value of deformation γ and thus the modulus of elasticity G are determined.

τ = G·γ,(1)

To determine the complex modulus of deformation, the mode of rotational sinusoidal oscillations τ sin(ωt) with increasing amplitude τ in the range from 0.1 Pa to the oscillation stall (about 200–250 Pa) was created. The deformation γ also occurs according to sinusoidal dependence with a phase lag γ sin(ωt + δ); the device outputs the phase shift δ between τ and γ during the oscillatory motion of the disk and the components of the complex modulus G*: elasticity modulus G′ and viscosity modulus G″.

τ = G*·γ,(2)

**Figure 12:**
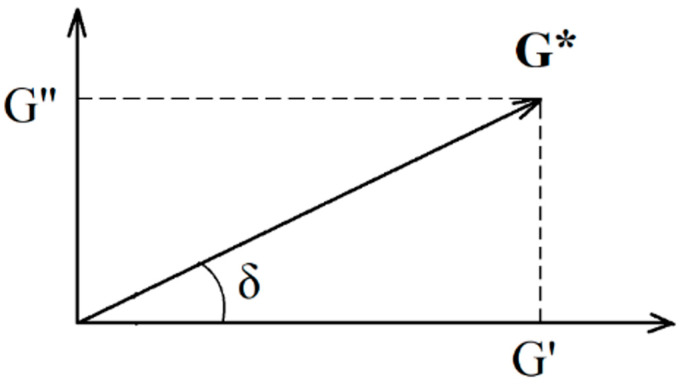
The components of the complex modulus G′ and G″ on the complex plane, where δ is the phase angle.

The complex module G* can be represented on the complex plane (Figure 12).

G* = G′ + *i*·G″ (3)

The magnitude of the complex modulus is as follows:

(4) $$\left|G*\right|=\sqrt{{G^{\prime }}^{2}+{G^{\prime \prime }}^{2}}$$

The linear measurement range (LMR) was determined by the rheometer program based on the measured data G′. Physically, this corresponds to the onset of significant loss of elasticity with increasing stress. In the graphs (Figure 4), this range is indicated by the vertical line, with values of τ = 48.8 Pa and τ = 44.8 Pa for FGS A345 and A523, for the hydrogel τ = 19.6 Pa.

The G″ and |η*| given in Table 10 correspond to these values τ, also given by the computer.

The modulus of elasticity G′ is the real part of the complex modulus of elasticity (Equation (3)) and is tabulated by the rheometer as a function of the shear stress values τ. This modulus displays the elastic properties of the medium.

The viscosity modulus (loss modulus) G″ is the value of the imaginary part of the complex modulus of elasticity and is tabulated by the rheometer as a function of shear stress values τ. This modulus displays the losses associated with viscous friction.

When the crossover point is reached (the intersection of lines G′ and G″), the body loses its elastic properties and becomes a fluid medium (liquid).

The rheological parameters of the studied compositions are presented in Table 10.

### 4.4. Filtration Studies

Filtration studies were conducted using an SMP-PS/FES-2P unit on three porous medium models: a slot model (nominal gap 0.04 cm) and two sandpack models, one with a Fores 20/40 proppant (Sandpack P) and the other with a disintegrated core (Sandpack DC). The volume of FGC injection is one-third of the pore volume. The exposure time is 1.5 h after the preparation of the composition. The technical characteristics of the unit are given in Table 11.

**Figure 13:**
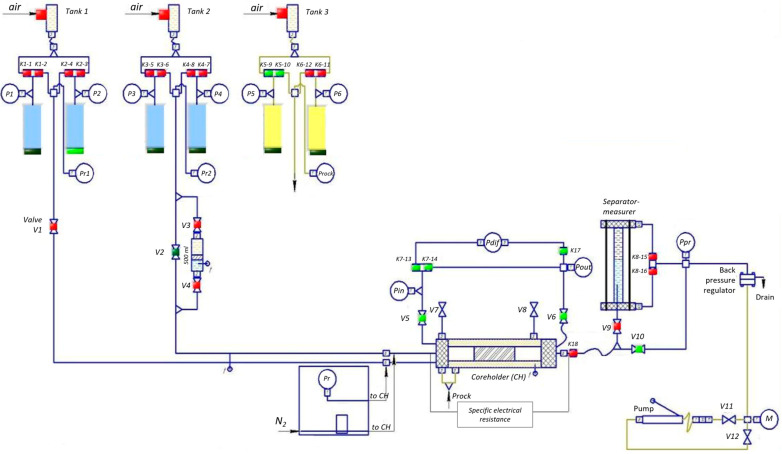
Schematic diagram of experimental unit SMP-PS/FES-2P.

The SMP-PS/FES-2P filtration unit (Figure 13) consists of the main unit (working table) and the electronics unit. The main unit contains the following:

- Core holder with an electric heater;
- The main hydraulic system, which performs the functions of formation fluids supply and determination of their volumes (the main system includes two subsystems corresponding to two fluid phases);
- An auxiliary hydraulic system designed to create rock (crimp) pressure;
- A system for creating back pressure during filtration;
- The sensors of the control and measurement system;
- The distribution gas combs of the pneumatic valve control system connected to the air line at 5.5–6.5 atm.

To measure and regulate the temperature, the system “thermocouples (thermoresistance)–meter-temperature regulator–computer–power relay-regulator–heating elements” is used, further separated into a temperature regulation system.

In the electronics block, the following are installed: HART-modem, the input-output unit of pressure sensors, the blocks (drivers) of the stepper motor control of high-pressure pump drives, power supply units, the input-output unit of pneumatic comb control, a personal computer system unit, a digital immittance meter, an electronic ultrasound unit and data acquisition board (oscilloscope) and a MOXA UPORT 1650-8 expansion board for eight COM ports.

The core samples were prepared for the experiments in accordance with the requirements of the Russian standard OST 39-195-86 [36]. The following thermobaric conditions were maintained during the experiments: pressure 15 MPa and temperature 60 °C. The filtration rate was set to 3 cm^3^/hour (excluding porosity). The main characteristics of the reservoir models are listed in Table 12.

### 4.5. The Method of Testing the Foam Gel Composition Using an Ideal Fracture Model

**Figure 14:**
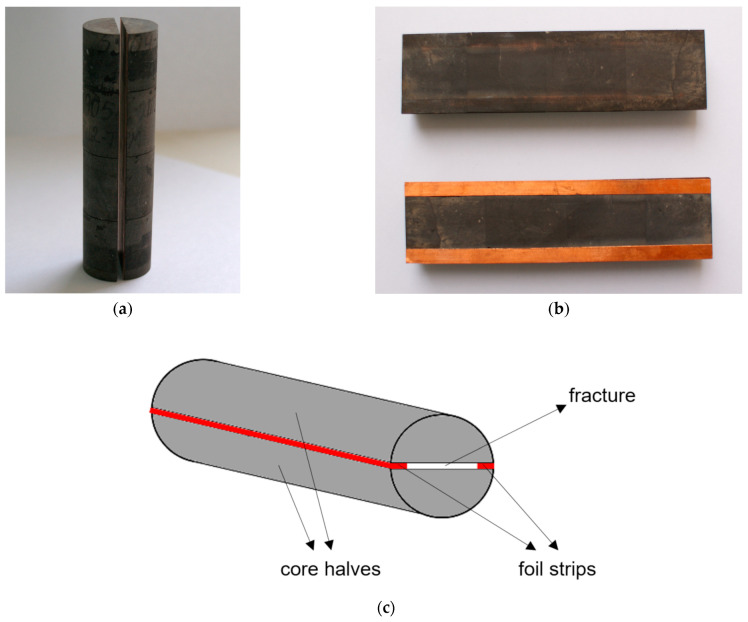
Photo of the ideal fracture model ((**a**)—photo of the sawn core; (**b**)—photo of the sawn halves of the core with glued foil strips; (**c**)—scheme of an ideal fracture) [37].

Natural low-permeability (less than 1 mD) core samples (represented by sandstone with preferential water wettability) were used to create an ideal fracture model (slot model—Figure 14), which satisfactorily reproduced the conditions of natural wettability. The entire procedure of slot model preparation is similar to that described in article [37].

Core samples were sawn lengthwise, extracted with an alcohol–benzene mixture, washed with bidistilled water from salts in Soxhlet extraction apparatuses and dried in a drying cabinet at a temperature of 105 °C. Strips of foil of the appropriate thickness were glued to one of the halves after polishing the mating surfaces of the slot model (to create a predetermined amount of fracture opening). The parameters of the ideal fracture model were as follows: length 11.2 cm; nominal gap (slot opening) 0.04 cm; orientation was horizontal.

The following procedure was used to conduct the tests. A model of an ideal fracture was placed in the core holder of the filtration unit, and the necessary thermobaric conditions were created. Next, fresh water was filtered until the pressure drop was stabilized (but not less than 10 pore volumes of the slot), and water permeability was measured before treatment with the chemical. Then, 10 cm^3^ of the tested composition was injected into the slot model (to completely replace the liquid in the slot space). After that, the system was shut-in under static conditions for at least a day. Then, the injection of the formation water model with a salinity of 20.5 g/L into the slot model was resumed.

### 4.6. The Procedure of Testing the Foam Gel Composition in the Sandpack Models

The experiment with the Sandpack P porous medium model determined the efficiency of the chemical for reducing the water phase permeability using a highly permeable model with a length of 15 cm (super reservoir). The residual resistance factor (Frr) as the criterion of efficiency of the water shutoff composition was calculated as the ratio of water permeability before the injection of the chemical to the water permeability after the injection at initial water saturation.

A sandpack model of a porous medium (Sandpack P) was prepared for the test, which simulates a super reservoir. Proppant Fores 20/40 brand was used as a filler, which was placed directly into the rubber crimping cuff of the core holder of the filtration unit.

Initially, the sandpack model of the formation was saturated with fresh water in the presence of effective pressure (crimping), and water permeability was determined. Then, the FGC was injected into the porous medium in the form of a separate rim, which is 0.3 pore volume. After that, the system was shut-in under static conditions for at least a day. The formation water model (with a salinity of 20.5 g/L) injection was resumed until the pressure gradient was fully stabilized, and Frr was calculated.

The method for conducting the experiment on a sandpack model with a disintegrated core (Sandpack DC) is similar to that for conducting the experiment on the Sandpack P model.

### 4.7. Field Test

**Figure 15:**
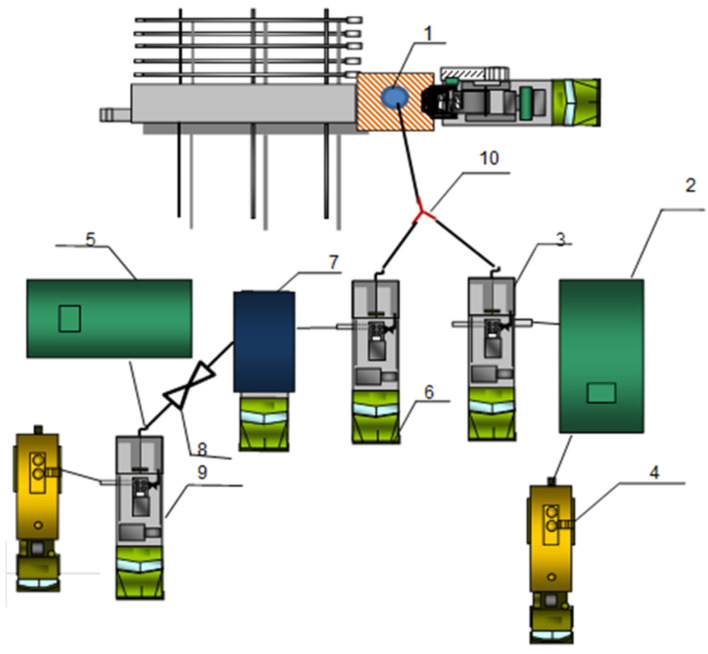
Scheme of equipment arrangement during injection of FGC (1—well; 2, 5—technological tank V = 20 m^3^; 4—tank trucks with fresh water; 3, 6, 9—cementing (pumping) unit CA-320; 7—mixing and mediating unit; 8—ejector; 10—tee).

**Figure 16:**
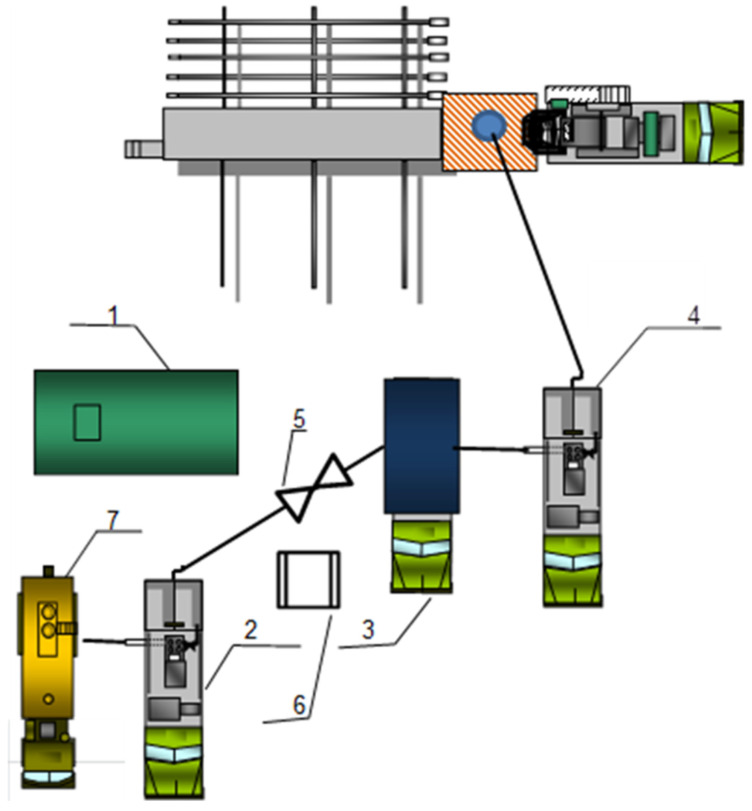
Scheme of equipment arrangement during hydrogel injection (1—technological tank with fresh water; 2,4—cementing (pumping) unit CA-320; 3—mixing and mediating unit; 5—ejector; 6—tray with hydrogel reagent; 7—tank truck with fresh water).

Schemes of special equipment deployment during gas shutoff operations at production wells are shown in Figure 15 (for FGC injection) and Figure 16 (for hydrogel injection).

FGC consists of two solutions (No. 1 and No. 2). For the preparation of working solutions in technological tanks (2) solution No 1 and (5) solution No 2, the estimated amount of fresh water is taken. The calculated amount of FGC components is then loaded sequentially and stirred for 3–4 h until complete dissolution. The preparation of solution No. 1 is performed using an ejector (8), which connects the cementing unit (9) and the mixing and mediating unit (7). FGC solutions are pumped simultaneously (with the same flow rate) using two cementing units CA-320 (6) and (3) through a tee (10).

The process of hydrogel injection is as follows. In the technological tank (1), for the preparation of hydrogel composition, the estimated amount of fresh water is taken. In the discharge line between unit CA-320 (2) and mixing and mediating unit (3) through the installed jet pump for chemical reagent dosing, ejector (5), the calculated amount of hydrogel reagent is dosed. The time of reagent dissolution after filling the whole volume of hydrogel into the mixing and mediating unit is 1–2 h. The prepared hydrogel solution is pumped using the cementing unit CA-320 (4). Reagent pumping is performed with a closed annular space. After pushing those with water, the well is closed for 24 h for the reaction and crosslinking of the injected reagents.

## Figures and Tables

**Table 1:** The results of the oscillation test of FGC.

Tested Composition	Linear Measurement Range, Pa	G′, Pa	G″, Pa	Crossover, Pa	G′ = G″, Pa	η′ = η″, Pa·s ^1^
FGC A345	48.84	35.85	8.093	285.7	3.744	0.596
FGC A523	44.81	34.38	6.837	295.0	4.958	0.789

^1^ η′ = G′/ω, η″ = G″/ω.

**Table 2:** Residual resistance factor of a foam polymer system in porous media of various structures.

Test No.	Porous Medium	Water Permeability, µm^2^		Composition	Frr, u.f.
		Before the Injection of the Composition	After the Injection of the Composition		
1	Slot model	25.71	0.42	PAM (A523) 0.6% + sodium nitrite 8% + ammonium chloride 7% + chromium acetate 0.3% in fresh water	61.21
2	Sandpack P	27.79	0.84		33.08
3	Sandpack DC	11.4 × 10^−3^	2.15 × 10^−3^		5.30

**Table 3:** Liner dimensions at wells W1, W2 and W3.

Well No.	Liner Diameter, mm	Liner Wall Thickness, mm	Liner Length, m
W1	114.3	7.4	1282.38
W2	114.3	7.4	743.08
W3	114.3	7.4	884.34

**Table 4:** Design of staged treatment of two intervals in well W1.

Parameter	Stage 1	Stage 2
Treatment interval, m	1680–1760	1280–1370
Bridge plug setting depth (isolation of leakage interval), m	1826	1475
Interval of perforation of special openings, m	1702–1703	1285–1286
Mule shoe depth, m	1690	1275
FGC injection volume, m^3^	30	70
Hydrogel injection volume, m^3^	5	15
Injection pressure, atm	≤60	

**Table 5:** Performance parameters of well W1 before and after treatment.

Parameter	Qliq, m^3^/Day	Qoil, t/Day	WC, %	Pbottom, Atm	Hdyn, m	Qg.c., m^3^/Day	GOR, m^3^/t
Before treatment	8.1	6.8	11	32.0	not determined	115,002	16,941
After treatment	4.0	2.9	40	26.9	557	0	29
As of 1 November 2022	3.1	2.6	12	28.8	802	0	29
As of 1 September 2023	1.1	0,9	14	36.2	640	68	109

**Table 6:** Design of staged treatment of two intervals in well W2.

Parameter	Stage 1	Stage 2
Treatment interval, m	1718–1906	1530–1718
Bridge plug setting depth, m	1906	1718
Mule shoe setting depth, m	1718	1530
FGC injection volume, m^3^	50	50
Hydrogel injection volume, m^3^	10	5
Injection pressure, atm	≤60	

**Table 7:** Performance parameters of well W2 before and after gas shutoff treatment.

Parameter	Qliq, m^3^/Day	Qoil, t/Day	WC, %	Pbottom, Atm	Hdyn, m	Qg.c., m^3^/Day	GOR, m^3^/t
Before treatment	12.7	9.7	19.7	53	426	43947	4576
After treatment	10.0	8.5	10.3	46.9	376	0	29
As of 1 November 2022	12.0	10.0	12.0	42.4	603	0	29
As of 1 September 2023	13.0	10.3	16.0	42.7	633	6329	642

**Table 8:** Design of treatment of well W3.

Parameter	Numerical Value
Treatment interval, m	1729–1875
Bridge plug setting depth, m	1877–1874
Combined tubing depth, m	1736
Technological packer depth, m	1726–1729
FGC injection volume, m^3^	100
Hydrogel injection volume, m^3^	20
Injection pressure, atm	≤90

**Table 9:** Performance parameters of well W3 before and after gas shutoff treatment.

Parameter	Qliq, m^3^/Day	Qoil, t/Day	WC, %	Pbottom, Atm	Hdyn, m	Qg.c., m^3^/Day	GOR, m^3^/t
Before treatment	8.7	8.0	2.6	36.9	628	56,393	7045
After treatment	15.0	12.0	15.3	37.5	676	3901	354
As of 21 September 2023	12.6	10.1	15.0	34.8	678	53,716	4274

**Table 10:** The rheological parameters.

Composition	The Linear Measurement Range				The Crossover Point of the Modules G′ and G″		
	LMR, Pa	G′, Pa	G″, Pa	|η*|, Pa	Cr, Pa	G′= G″, Pa	η′ = η″, Pa·s
1	48.84	35.85	8.093	4.142	285.7	3.744	0.596
2	44.81	34.38	6.837	4.745	295.0	4.958	0.789
3	19.64	45.26	15.88	7.633	74.36	8.751	1.393

**Table 11:** The technical characteristics of the SMP-PS/FES-2P unit.

Parameter	Numerical Value
Linear length of the core model, mm	25 to 300 mm
Maximum rock pressure, MPa	70
Maximum reservoir pressure, MPa	55
Maximum effective pressure, MPa	60

**Table 12:** Main characteristics of reservoir models.

Test No.	Porous Medium	Length, cm	Diameter, cm
1	Slot model	11.20	3.00
2	Sandpack P	15.00	3.00
3	Sandpack DC	15.00	3.00

## Data Availability

The data presented in this study are available on request from the corresponding author.
